# Post-COVID Decline in Social Engagement and Cognitive Function Among Chinese Older Adults

**DOI:** 10.1093/geroni/igaf122.1450

**Published:** 2025-12-31

**Authors:** Wei Zhang

**Affiliations:** University of Hawaiʻi at Mānoa, Honolulu, Hawaii, United States

## Abstract

**Purpose:**

This study examines the impact of reduced social engagement on cognitive function among older adults in China during the COVID-19 pandemic, with a focus on gender and urban-rural differences.

**Methods:**

Using data from the China Health and Retirement Longitudinal Study, we employed OLS regression models to assess changes in cognitive function before and after the pandemic, focusing on how these changes are linked to changes in social engagement, which was measured across a variety of activities, including both physical and cognitively stimulating tasks.

**Findings:**

Significant cognitive declines were observed across all subgroups, with urban females experiencing the greatest reduction. Reduced participation in moderate physical activities appeared to have a protective effect, while decreased engagement in cognitively stimulating activities, such as playing games, was associated with greater cognitive decline.

**Conclusions:**

The study highlights the importance of maintaining social engagement, particularly in cognitively stimulating activities, to mitigate cognitive decline among older adults. The observed differences in cognitive health outcomes by gender and urban-rural status suggest a need for targeted interventions to support older populations in the post-pandemic context.

